# Tet Transgene Activation is Disrupted in Lipogenic Triple Negative Breast Cancer Cells

**DOI:** 10.1021/acssynbio.4c00851

**Published:** 2025-07-08

**Authors:** Ashley Townsel, Yifei Wu, Maya Jaffe, Cara Shields, Karmella A. Haynes

**Affiliations:** † Department of Biology, 1371 Emory University, Atlanta, Georgia 30322, United States; ‡ Wallace H. Coulter Department of Biomedical Engineering, 1371 Emory University, Atlanta, Georgia 30322, United States

**Keywords:** transgene, epigenetics, metabolism, lipogenesis, breast cancer, cell line

## Abstract

A critical challenge for mammalian cell engineering is the unexpected response of transgenes to native transcriptional regulation pathways. One transgene can show different levels of expression at different genomic sites, in different cell types, and under different growth conditions. Collisions between transcription and DNA replication, heterochromatin encroachment, and viral defense have been linked to transgene silencing. In this study, we identify fatty acid metabolism as another mediator of transgene behavior. Adipocyte secretome-induced lipogenesis in epithelial breast cancer cells was accompanied by the loss of expression from a Tet-TA regulated *pCMV-AmCyan* reporter transgene. Transcription profiling showed activation of lipid droplet biosynthesis, and repression of loci across the genome, consistent with the idea that lipogenesis affects the availability of substrates and cofactors for global chromatin remodeling. Preinduction of *pCMV* prevented full silencing during lipogenesis. Our results provide new insights into the influence of shifting metabolic states on transgene behavior.

## Graphical abstract

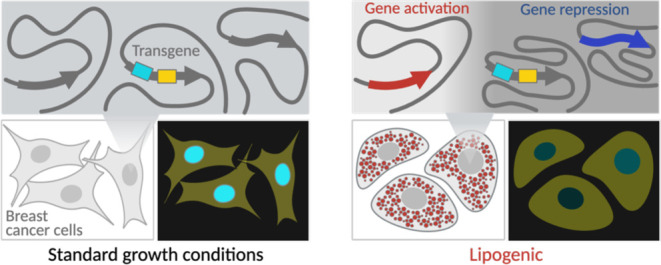

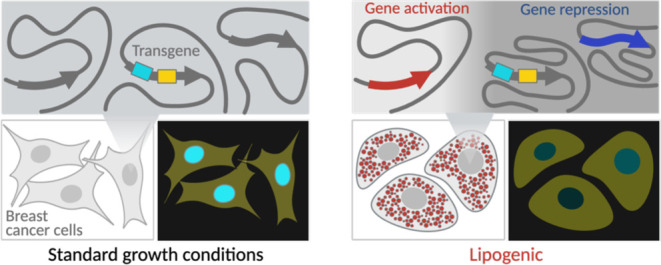

## Introduction

Transposon systems are indispensable for cell engineering, as they allow the stable integration of transgenes into the host genome, ensuring heritable expression in proliferating cells. However, transgene expression is not entirely predictable. Competition with endogenous gene expression for transcriptional machinery can lead to transgene silencing.^1^ CpG island methylation and histone deacetylase activity can destabilize histone acetylation-dependent transcription.^2,3^ Furthermore, the activity of histone methyltransferases at endogenous silenced heterochromatin can spread to nearby transgenes.^4,5^ These repressive mechanisms force transgene regulation to default to native chromatin states. Silencing can also result from innate defense systems that repress transcription and translation of transposon components.^6–8^

Environmental cues and cellular metabolism also influence epigenetic states. Adipocyte secreted factors (ASFs) activate signaling pathways including PI3K/AKT/mTOR,^9,10^ NF-κB,^11^ JAK/STAT3,^12^ hypoxia-induced HIF-1α,^13^ and MAPK/ERK,^14^ leading to the activation of genes that encode metabolic enzymes that drive fatty acid beta oxidation, the TCA cycle, and the salvage pathway. These processes affect levels of substrates used in chromatin remodeling, such as acetyl-CoA, alpha-ketoglutarate (α-KG), flavin adenine dinucleotide (FAD+), and nicotinamide adenine dinucleotide (NAD+).^15–18^ Acetyl-CoA is crucial for histone acetyltransferases (HATs), which support active gene transcription. A close relationship between histone acetylation and acetyl-CoA levels has been determined in human normal and cancer cells.^19^ Inhibition of acetyl-CoA carboxylase (ACC1), which consumes acetyl-CoA during lipogenesis, results in increased global histone acetylation in yeast.^20^

Here, we report the transcriptional repression of a chromosomally integrated transgene, *pSBtetTA-YP_CFP*, in ASF-stimulated, lipogenic breast cancer epithelial cells. This expression system allowed us to observe the activities of a doxycycline-inducible promoter (*pCMV*) regulated by a VP16 fusion regulator (Tet-TA) and a separate constitutive promoter (*pRPL13a*).^21^ Imaging and flow cytometry showed that Tet-TA regulated expression is strongly repressed in lipogenic breast cancer cells but not in HEK293 cells. To determine how the lipogenic state might affect transcription initiation and active transcription, we stimulated lipogenesis before, during, and after doxycycline-induced transcriptional activation. Inducing the lipogenic epigenetic state prior to transcriptional activation blocked transgene expression more effectively than when lipogenesis was induced after transcriptional initiation.

## Results

### Adipocyte Conditioned Media Stimulates Lipid Droplet Synthesis and Gene Expression Changes in TNBC BT-549 Cells

To model signaling from adipocytes to breast cancer cells, we treated breast epithelial BT-549 cells with adipocyte conditioned medium (ACM) (Figure 1A). After 2 days, we observed a striking accumulation of lipid droplets in the cytoplasm of most cells, whereas lipid droplets were barely visible in cells grown in unconditioned medium (UCM) (Figure 1B). These results are consistent with other reports where adipocyte-secreted factors induce lipid accumulation in breast cancer cells^22,23^ and are thought to serve a pro-oncogenic role.

**Figure 1:**
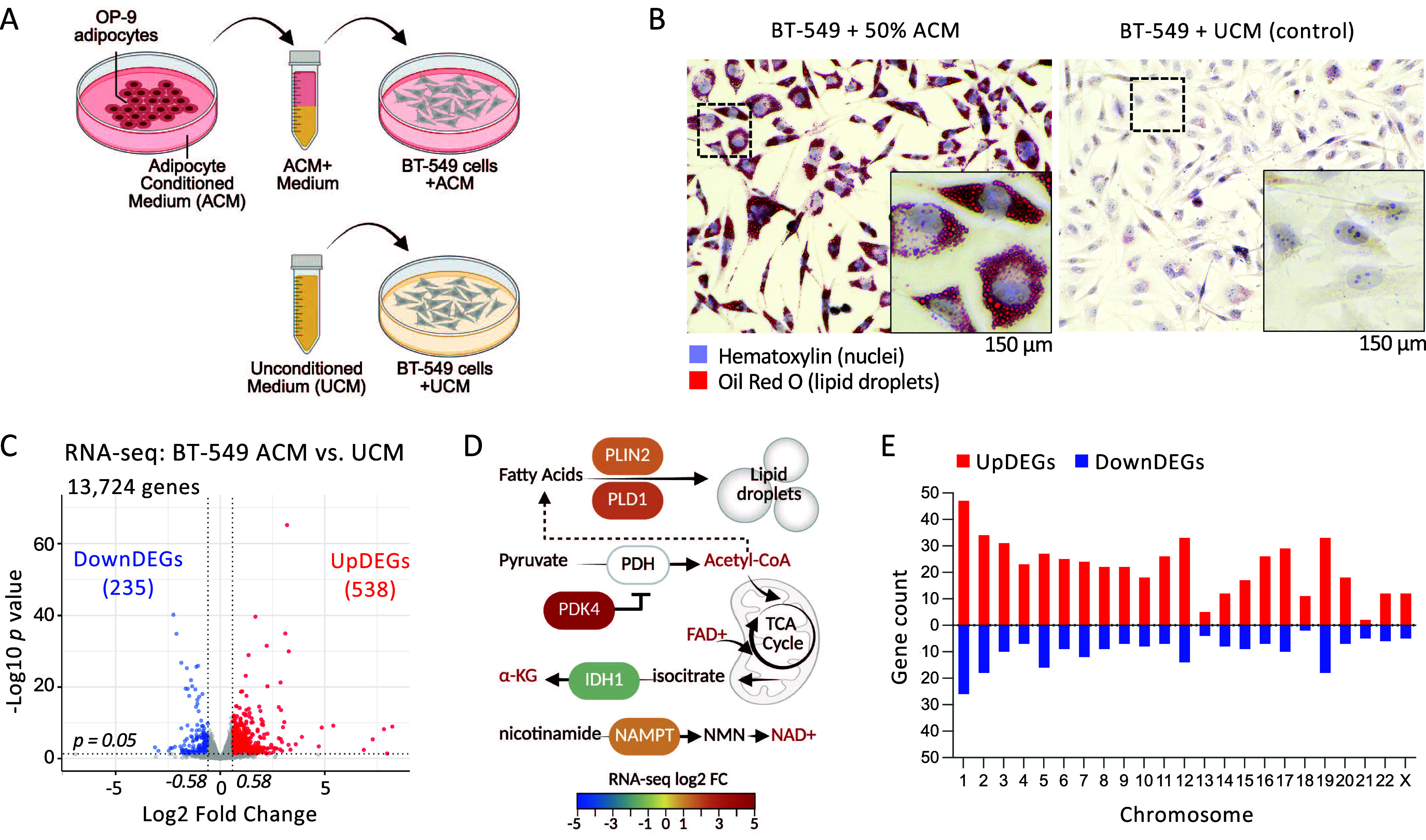
Adipocyte-conditioned media induces lipid droplet formation and gene expression changes in BT-549 cells. (A) OP-9 mouse adipocytes are differentiated *in vitro*, and the adipocyte conditioned medium (ACM) is collected and added to cultured BT-549 cells. (B) Oil Red O staining of lipid droplets. (C) RNA-seq expression profiling to identify differentially expressed genes (DEGs). (D) Metabolic functions associated with select DEGs. (E) Genomic distribution of DEGs. Illustrations (A, D) were generated in BioRender.

Lipogenic cells use acetyl-CoA to synthesize fatty acids.^24^ Acetyl-CoA is also required for histone acetylation to support gene transcription. Therefore, we investigated how increased lipogenesis impacts gene regulation. Comparative RNA-seq transcription profiling of BT-549 cells cultured in ACM versus UCM identified 235 repressed genes (DownDEGs), and 538 activated genes (UpDEGs) (fold change ± 1.5, log2 FC ± 0.58, *p* ≤ 0.05, Figure 1C). UpDEGs were associated with responses to chemical stimuli, cytokines, and lipids, storage of lipids, cell migration, and cell proliferation (Supplemental Figure S1A). Specific UpDEGs that regulate lipid droplet biosynthesis include *PLD1* (phospholipase D1)^25^ and *PLIN2* (perilipin-2)^26^ (Figure 1D). Other metabolic enzyme UpDEGs included *PDK4* (pyruvate dehydrogenase kinase 4)^27,28^ and *NAMPT* (nicotinamide phosphoribosyltransferase) which impact oxidative phosphorylation and NAD biosynthesis, respectively.^18^ *IDH1* (isocitrate dehydrogenase 1) which converts isocitrate into α-KG^29^ was downregulated. DownDEGs appeared at every chromosome (Figure 1E), suggesting widespread silencing. Many DownDEGs encode proteins associated with cell adhesion and immune response, consistent with enhanced migration and evasion of the immune system (Supplemental Figure S1A). However, the unexpected downregulation of sterol, cholesterol, and secondary alcohol biosynthesis genes, which contribute to lipid storage, might reflect feedback inhibition of SREBP-mediated transcription.^30^

To investigate why gene subsets show such distinct responses to the same stimulus (ACM), we compared transcription factor binding motifs in the promoters of UpDEGs and DownDEGs (Supplemental Figure S1B-D). Promoter motifs that were significantly enriched in UpDEGs and appeared in the top ten most activated genes are targeted by ATF4, NFKB1, SP1, and MEF2A, which are known activators of stress response, inflammatory, and metabolic adaptation programs. This is consistent with stimulation by adipocyte-secreted cytokines and metabolites through the NF-κB and PI3K/Akt pathways,^11,31^ and by adipocyte-induced metabolic stress through the integrated stress response (ISR).^32^ The DownDEGs showed enrichment of binding motifs for transcription factors associated with differentiation (ALX4, ESX1, HOX, MYF6, and PBX2) and tumor suppressor function (HIC1 and MAX) which are not strongly linked with adipocyte response. A motif recognized by SP1 and SP3 was highly represented, and SP3 can compete with SP1 to repress transcription.^33^ The DownDEGs may be subject to insufficient activation in the absence of adipocyte-induced signaling, as well as active repression (e.g., by SP3), potentially compounded by chromatin reorganization.

Metabolic flux alterations during lipogenesis could contribute to chromatin modifications that suppress the transcription of the DownDEGs. PDH inhibition by PDK4 potentially limits acetyl-CoA availability for the TCA cycle and histone acetyltransferases (HATs). Reduced TCA cycle activity may decrease α-KG production, impairing α-KG-dependent chromatin remodelers such as histone H3K9 and H3K27 demethylases.^34^ Repressive chromatin might be reinforced by NAMPT, which produces NAD+ required for Sirtuin activity^35,36^ (log2 FC = 0.95, *p* < 0.001). Additionally, slower TCA cycling might conserve FAD+, possibly enhancing the activity of enzymes that deposit repressive methyl marks on DNA and histones. While these speculations are consistent with known metabolic and epigenetic pathways, further studies are needed to determine the direct links between lipid metabolism and chromatin remodeling.

### Tet-TA Regulated Transgene Expression Is Perturbed in Lipogenic TNBC Cells

To investigate the impact of the lipogenic state on transgene behavior, we generated stable transgenic cell lines using a customizable sleeping beauty transposon-based reporter (Figure 2A) that allows semirandom insertion across the genome.^21^ Expression of Venus yellow fluorescent protein (YFP), a Tet-3xVP16 transcriptional activator (Tet-TA), and puromycin are driven by a constitutive *RPL13a* promoter. Expression of AmCyan fluorescent protein (CFP) tagged with a nuclear localization sequence (CFP-NLS) is regulated by doxycycline-bound Tet-TA, which binds to 7 copies of a 19-bp TetO sequence upstream of a CMV core promoter (Figure 2A). Nonlipogenic cells grown in UCM showed dim to very bright nuclear CFP after treatment with 1.0 μg/mL doxycycline for 2 days (Supplemental Figure S2). Cells treated with ACM and 1.0 μg/mL doxycycline showed reduced CFP-NLS (Supplemental Figure S2), and repression was more pronounced with 0.5 μg/mL doxycycline (Figure 2B). Low CFP-NLS signal corresponded with high lipid droplet content.

**Figure 2:**
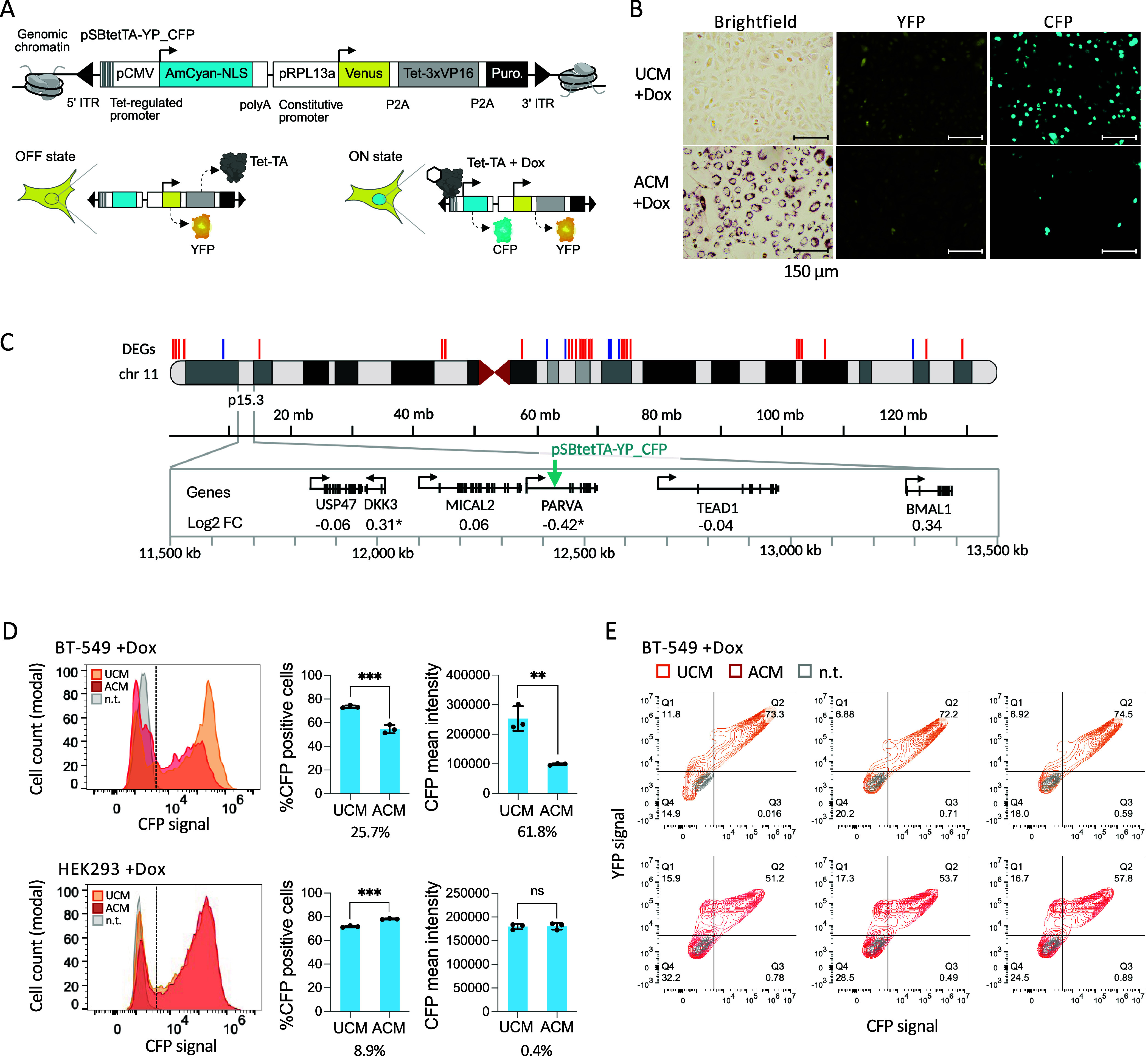
Adipocyte conditioned medium induces repression of a transgene in BT-549 breast cancer cells. (A) Schematic of transgene *pSBtetTA-YP_CFP* (generated in BioRender). (B) Fluorescence imaging of CFP-NLS expression in BT-549 cells. (C) Map of all DEGs (up = red, down = blue) on chromosome 11. The enlarged map shows 2.0 Mb from cytoband p15.3 containing the *pSBtetTA-YP_CFP* insertion site (teal arrow) in *PARVA* and surrounding genes with RNA-seq values shown. *p* ≤ 0.01*. (D) Flow cytometry analysis of nontransgenic (n.t.) control and transgenic BT-549 and HEK293 cells treated with doxycycline (dox) and UCM or ACM. Histograms show one sample from each condition. Bar charts show means of three replicate wells per condition, percent differences between mean UCM and ACM values, standard deviation, and unpaired *t* test values: *p* ≤ 0.05*, 0.01**, 0.001***, or not significant (ns). (E) Contour plots of YFP and CFP flow cytometry signals from all replicates.

To identify the transgene insertion site, we sequenced a genomic fragment that contained a junction between the *pSBtetTA-YP_CFP* 5′ inverted terminal repeat (5′ ITR) and adjacent chromosomal DNA (Figure 2C and Supplemental Figure 3). The sequence mapped to an intron of *PARVA*, which showed slight, significant downregulation in ACM-treated cells. Genes within the surrounding 2.0 Mb showed modest changes in either direction (log2 FC −0.06 to 0.34). Therefore, transgene silencing can occur in an environment where slight repression or no change of genes occurs in response to lipogenesis.

Flow cytometry analysis showed that 72 h ACM treatment reduced CFP-positive cells by 25.7%, and the mean fluorescence intensity of this population was reduced by 61.8% compared to the UCM samples (Figure 2D, top). To determine if repression might require surrounding chromatin, we tested the effect of ACM on transgene expression prior to stable integration. In YFP-positive transfected cells, CFP frequency was reduced by 29.7%, but the mean signal was not affected (Supplemental Figure S4A). This result suggests that pCMV activation was partially inhibited on episomal DNA, but dampening of transcription depends upon transgene integration and chromatin remodeling at the insertion site. To determine the generalizability of this phenomenon for other transgenic contexts, we tested lentiviral inserts with reporters driven by constitutive promoters:^37^ *pCMV-luciferase* and *pSV40-GFP* in MDA-MB-468 and MDA-MB-231 TNBC cells, and *pPGK-RFP* in MCF-10A immortalized breast epithelial cells (Supplemental Figure S4B, C). We observed no significant change in luciferase expression, and a loss of GFP expression in MDA-MB-468 but not in MDA-MB-231. The difference in *pSV40-GFP* response could be due to cell line, insertion site, or transgene copy number-dependent effects. In MCF-10A, ACM treatment lowered the expression of RFP, suggesting that *pPGK* is also sensitive to the lipogenic epigenetic state in a non-TNBC breast cell line.

To determine if repression can occur in cells that do not become lipogenic in response to ACM, we tested HEK293 cells. No changes in cell morphology or lipid droplet content were observed in HEK293 cells treated with ACM (Supplemental Figure S5A). Transgenic HEK293 cells were generated and subjected to the same experiments as for BT-549. CFP-NLS expression showed either modest changes (less than 10%) or no significant changes in one cell line (Figure 2D, bottom) and in additional clonal isolates (Supplemental Figure S5B), suggesting that ACM-induced lipogenesis and epigenetic repression are cell-type dependent.

It is possible that loss of Tet-TA via epigenetic silencing of *pRPL13a* contributes to the loss of CFP. To investigate this idea we compared *pRPL13a*-regulated YFP protein expression (Figure 2A), in the UCM + Dox and ACM + Dox samples. We observed subpopulations of cells (15.9% - 17.3%) that were YFP-positive and CFP-NLS-negative (Figure 2E). We also compared transcript levels for Tet-TA, YFP, and CFP-NLS with reverse transcription followed by quantitative PCR (RT-qPCR). In UCM + Dox and ACM + Dox treated cells, average Tet-TA and YFP levels were similar, while CFP-NLS transcript levels were reduced in ACM-treated cells (Figure 3, and Supplemental Figure S6). These results suggest that *pCMV* is repressed by the lipogenic epigenetic state even when Tet-TA is present, and that the inducible *pCMV* promoter is more strongly repressed than the constitutive *pRPL13a* promoter.

**Figure 3:**
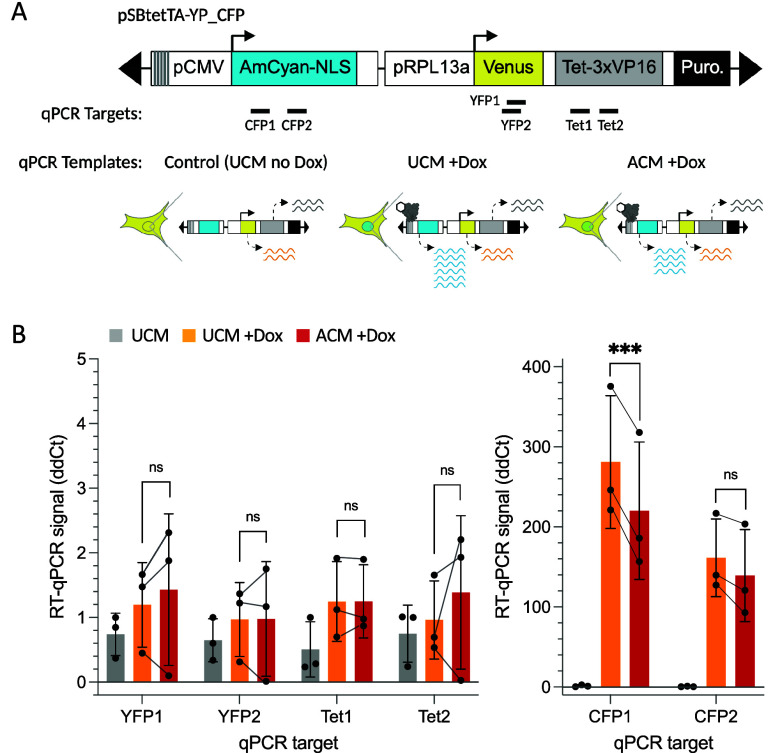
RT-qPCR analysis of transgene mRNA levels in uninduced (UCM), and doxycycline-induced (+Dox) cells treated with UCM or ACM. (A) The map shows the qPCR target sites: the CFP region of CFP-NLS (CFP1, CFP2), YFP (YFP1, YFP2), and the Tet region of Tet-TA (Tet1, Tet2). (B) Three cell samples per condition were used to generate cDNA templates for qPCR. GAPDH normalization is described in Materials and Methods. P-values were calculated with a paired two-tailed *t* test: *p* ≤ 0.05*, 0.01**, 0.001***, or not significant (ns).

### Characterization of Other Potential Repressive Sites in Polygenic Cells

To determine if ACM-induced transgene silencing could be observed at other loci, we tested a nonclonal population of transgenic BT-549 cells. *pSBtetTA-YP_CFP* is expected to undergo semirandom insertion at dispersed TA-dinucleotides, an estimated 30,000 sites in the human genome.^21^ Puromycin-selected nonclonal cells were seeded at low density, allowed to grow into small colonies to form isogenic clusters, and treated with ACM or UCM and 0.5 μg/mL doxycycline for 3 days (Figure 4A). The UCM-treated cells showed an approximately positive correlation between YFP and CFP signals. The ACM-treated cells showed a similar range of YFP values, however CFP was reduced. Next, cells were expanded under selection, subjected to the same treatments, and analyzed single cells by flow cytometry using YFP as a positive marker for transgenic cells. We saw a reduction in CFP signal among the YFP-positive and CFP-NLS-positive subpopulation, suggesting that ACM-induced repression occurs at insertion sites throughout the genome (Figure 4B). We also performed this experiment with TNBC cell lines of genome ancestries other than BT-549 cells (63% South European).^38^ HCC1806 (81% African) and MDA-MB-453 (60% North European) YFP/CFP-positive cells also showed a reduction in CFP signal with ACM treatment, suggesting the generalizability of ACM-induced repression across cell lines. These results suggest that many insertion sites, possibly outside of *PARVA*, show ACM-induced transgene silencing in different genetic backgrounds.

**Figure 4:**
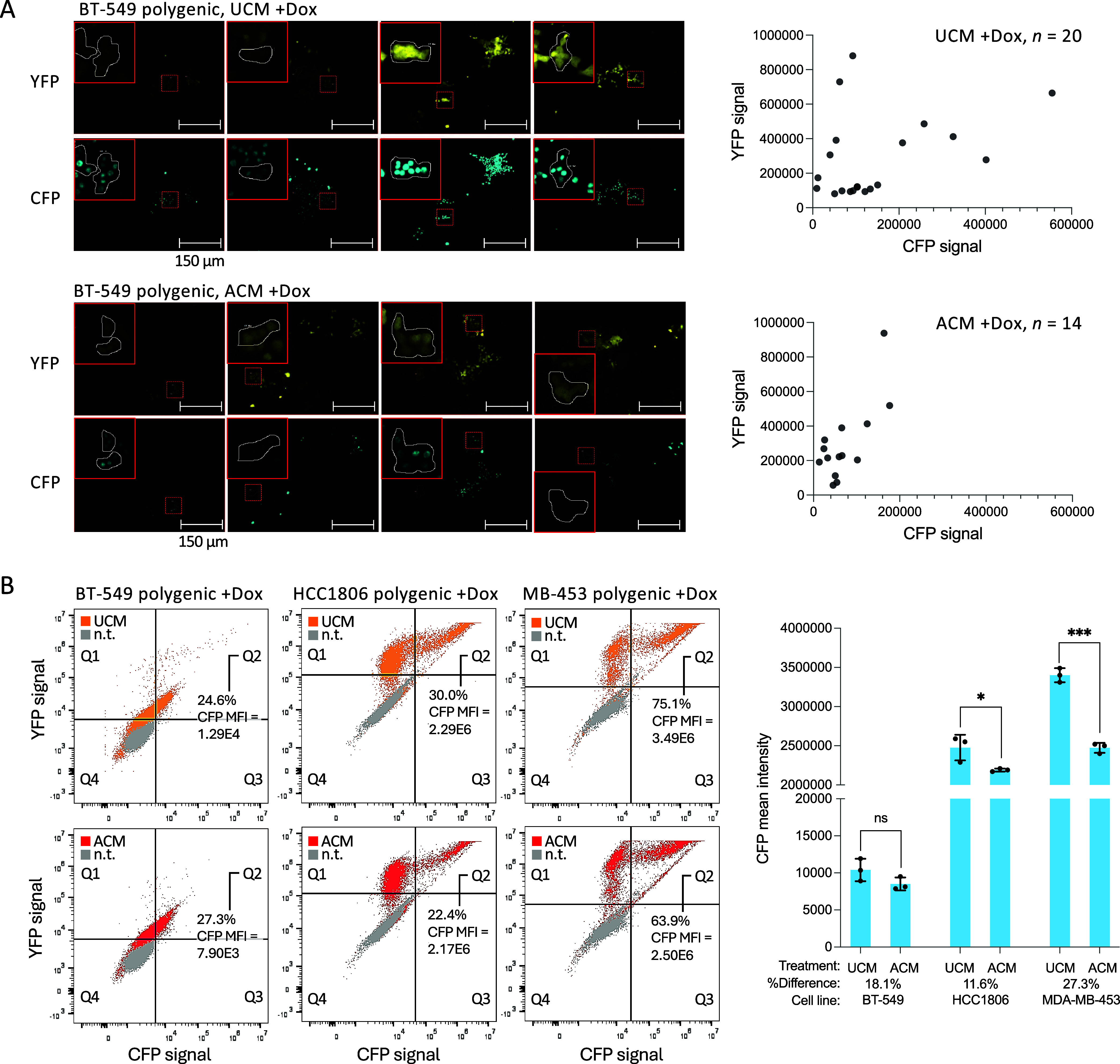
Adipocyte conditioned medium affects expression of transgenes in polygenic triple negative breast cancer cells. (A) Analysis of colonies grown from freshly transfected BT-549 cells prior to the isolation of an isogenic clone. YFP and CFP signal is the integrated density of areas containing 3 to 7 adjacent YFP-positive cells. Images of representative regions are shown above each plot. (B) Flow cytometry analysis of nontransgenic (n.t.) control and polygenic BT-549, HCC1806, and MDA-MB-453 cells treated with doxycycline (dox) and UCM or ACM (∼10,000 to ∼15,000 cells per sample). Scatter plots show one representative sample per condition. The bar chart shows mean CFP intensity of three replicate wells, percent differences between mean UCM and ACM values, standard deviation, and unpaired *t* test values: *p* ≤ 0.05*, 0.01**, 0.001***, or not significant (ns).

### Activation of *pCMV* Prior to ACM Treatment Protects the Transgene from Full Repression

Our results with the isogenic BT-549 cell line showed different effects of the lipogenic state on each of the two promoters in *pSBtetTA-YP_CFP*. One key difference between the promoters is that *pRPL13a-YFP* is already active before ACM treatment, whereas *pCMV-CFP-NLS* is inactive until Tet-TA binding is induced by doxycycline. To investigate the sensitivity of inducible versus constitutive transcription to the lipogenic state, we carried out stepwise treatments. We treated cells with ACM for 1 day, and then activated *pCMV-CFP-NLS* with doxycycline (Figure 5A, Treatment 1). Data from our previous ACM and doxycycline cotreatment experiments were used for comparison (Treatment 2). To determine if active transcription protects *pCMV-CFP-NLS* from repression, we treated cells with doxycycline before treating the cells with ACM (Treatment 3). We waited at least 48 h after ACM treatments before imaging the cells, which we determined was sufficient for fluorescent protein degradation after transcriptional silencing^39^ (Supplemental Figure 7).

**Figure 5:**
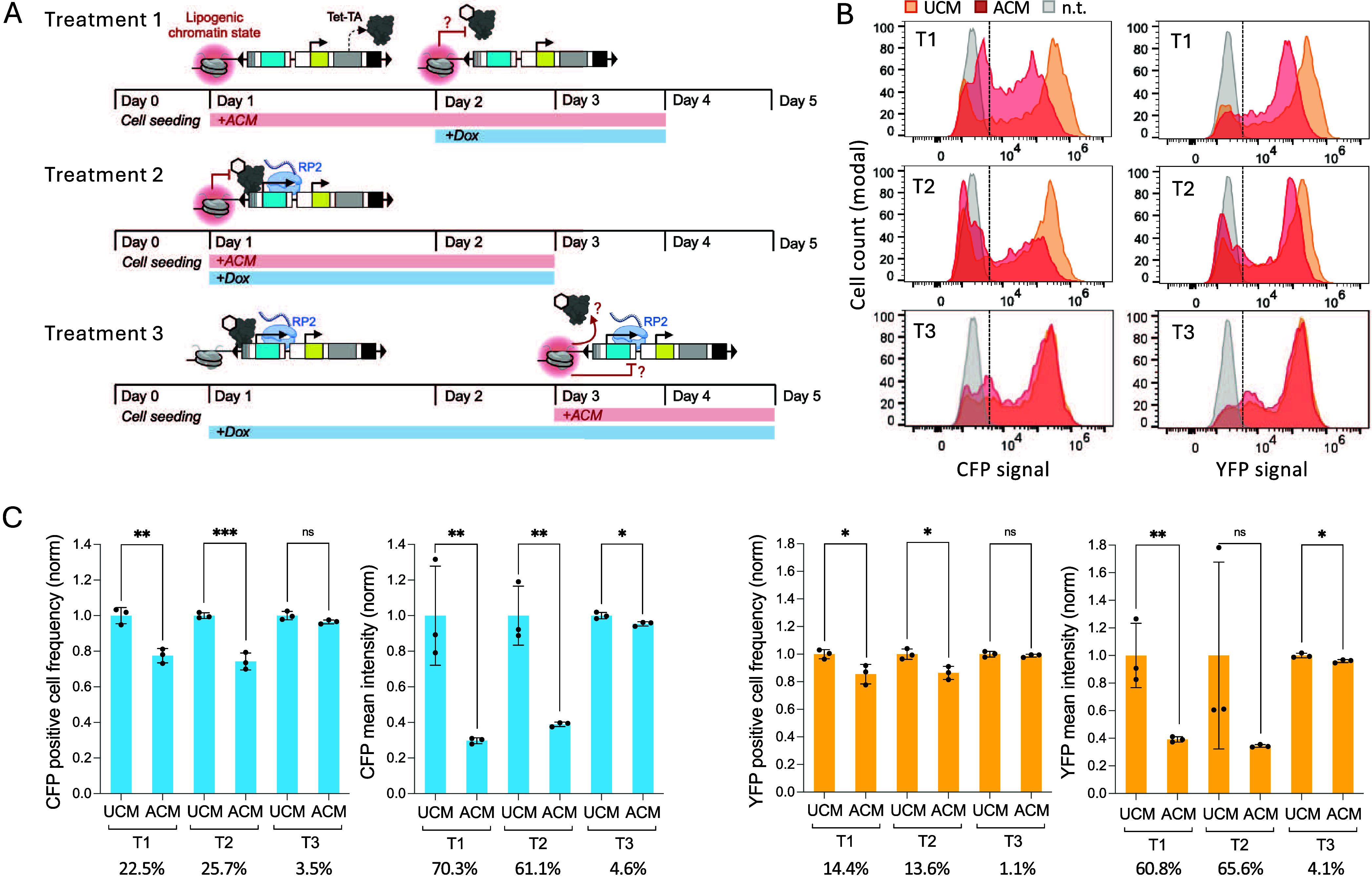
Comparison of lipogenic epigenetic repression of*pSBtetTA-YP_CFP* before, during, or after *pCMV* activation. (A) Experimental design. Controls were included in each treatment (T1, T2, and T3), where the media was replaced with fresh UCM instead of ACM. (B) Flow cytometry quantification of CFP and YFP expression in transgenic and nontransgenic (n.t.) BT-549 cells. (C) Bar charts show values normalized by the mean of three UCM replicates within each group (T1, T2, or T3), percent differences for mean UCM versus mean ACM, standard deviation (black bars), and unpaired *t* test values: *p* ≤ 0.05*, 0.01**, 0.001***, or not significant (ns).

Preinitiation of the lipogenic state resulted in a 22.5% decrease in the percentage of CFP-positive cells and a 70.3% decrease in the mean intensity of CFP (Figure 5B, C). Inducing the lipogenic state before *pCMV* activation (Treatment 1) resulted in a 8.9% stronger decrease in CFP mean intensity compared to simultaneous activation of lipogenesis and *pCMV* (Treatment 2). This suggests that repressive epigenetic mechanisms of the lipogenic state prevent transcriptional initiation, possibly by blocking access of Tet-TA to *pCMV*. In contrast, the preinitiation of *pCMV* activation (Treatment 3) resulted in an insignificant decrease in the percentage of CFP-positive cells and only a 4.6% decrease in mean CFP intensity.

YFP-positive cell frequency was less affected, showing smaller percent differences than the CFP-positive cell frequencies (Figure 5C). This is consistent with our observation that some ACM-treated cells that were CFP-negative were also YFP-positive (Figure 2E). In contrast, mean YFP signal intensity was reduced similarly to CFP with preinitiation of the lipogenic state (Treatment 1) and dual-initiation of the lipogenic state and *pCMV* activation (Treatment 2). It is worth noting that, removing the high YFP outlier in the Treatment 2 group would show that YFP mean intensity is less affected than for Treatment 1. The decrease in mean YFP intensity suggests epigenetic suppression of *pRPL13a* activity, which drives Tet-TA expression. Therefore, potentially lower concentrations of the Tet-TA regulator might contribute in part to the loss of CFP expression. These results suggest a mechanism where the initial activation of the promoter establishes a favorable chromatin environment, potentially marked by histone acetylation and persistent open chromatin that resists the effects of reduced acetyl-CoA and α-KG.

## Conclusion

This work demonstrates that transgene repression in cultured human breast cancer cells can occur as a consequence of a lipogenic metabolic state. Using a customizable sleeping beauty (pSB) based transgenic system, we observed lipogenesis-associated repression of a Tet-TA-targeted *CMV* core promoter. Genes that lack adipocyte-signal-activated promoters (e.g., SREBP factor targets) might be prone to epigenetic repression through the impairment of chromatin remodeling enzymes that rely on metabolites that are decreased during lipogenesis. The sensitivity of the transgene to the lipogenic epigenetic state may depend upon the initial activity level of the promoter. We showed that when lipogenesis was stimulated prior to activation of *pCMV* the expression level was decreased, whereas preactivation of the promoter prevented full repression.

A key limitation of our study is the use of a small set of model promoters, including inducible *TetO-pCMV*, constitutive *pCMV*, *pRPL13a*, *pPGK*, and *pSV40*. Other promoters may respond differently to metabolic states due to interactions with other transcription factors. Future work could explore the behavior of tissue-specific or inducible promoters to deepen insights into transgene behavior in the context of lipogenesis. Furthermore, our study did not measure chromatin accessibility with techniques like ATAC-seq or ChIP-seq, which could provide direct evidence of epigenetic changes at transgenes. Such analyses could correlate specific chromatin features with the observed transcriptional states.

Our results have implications for understanding transgene behavior in tissue microenvironments, and for optimizing *in vitro* cell culture to avoid transgene silencing. Monitoring epigenetic state changes via transgene reporter signals alongside tumor development could advance our understanding of how obesity-associated microenvironments drive cancer risk and progression. This approach could complement current research of adipose tissue’s influence in cancer as well as in metabolic and inflammatory diseases. Beyond cancer models, the shift between glucose-dependent and lipid-dependent energy production in response to cell signaling and nutrient availability is an essential process in industrially important transgenic cell systems including yeast, chinese hamster ovary (CHO) cells, and differentiating human stem cells. Therefore, scientists might consider monitoring the expression levels of key enzymes involved in lipogenesis when transgene expression becomes unstable in engineered cells.

## Materials and Methods

### Cell Culture

Media for each cell line is described in Supplemental Methods. Cells were grown at 37 °C, with 5% CO_2_ in a humidified incubator. 1X DPBS without calcium and magnesium (Corning #20-031-CV) and 0.25% trypsin-EDTA (Thermo #25200056) was used for cell washing and harvesting. For long-term storage, 1–2 × 10^6^ cells were frozen in 1 mL 10% DMSO in tet system approved FBS (Thermo #A4736401) at −80 °C for 2 days, then transferred to −150 °C (liquid nitrogen).

### Oil Red O Staining

Oil Red O working solution was prepared with 3 parts Oil Red O stock solution [0.35% g/mL Oil Red O (Sigma-Aldrich #O0625) in 100% isopropyl alcohol, passed through a 0.22 μm filter] and 2 parts distilled water, incubated at room temperature for 10 min. TNBC cells were seeded at 5 × 10^4^ cells/mL in a 6-well plate with 2 mL standard medium, grown overnight, then treated with either UCM or ACM for 2 days. The cells were washed with 1× DPBS, fixed with 4% paraformaldehyde (Thermo Fisher #J61899-AK) for 30 min, rinsed with water, and equilibrated with 60% isopropanol for 2 min at room temperature. Cells were stained with Oil Red O working solution at 37 °C for 10 min. Nuclei in nontransgenic cells were visualized by counterstaining with 1 mL Hematoxylin (Vector Laboratories #H-3404-100) for 30 s, and rinsing three times with water. Samples were covered with 1× DPBS and imaged at 200x.

### RNA-Seq

BT-549 cells were seeded at 1 × 10^6^ cells/mL in a 6-well plate and grown in UCM or ACM (3 replicates per condition) for 24 h. Adherent cells were lysed in the wells, and RNA was extracted and purified as instructed by the RNeasy Mini kit (Qiagen #74104). Frozen RNA (−80 °C) was submitted to Novogene for library preparation (polyA-enriched) and next generation sequencing (paired-end, 150 bp, Q30 ≥ 85%). Raw reads were trimmed with Trim Galore and aligned via seed searching using STAR^40^ to GRCh38/hg38. mRNA levels were calculated with RSEM^41^ and differential expression analysis was performed with DESeq2,^42^ using a negative binomial distribution algorithm. The volcano plot was generated with RStudio (version 4.4.1). Transcription factor binding sites were identified with the Transcription Factor Target Gene Database search tool (TFBS, https://tfbsdb.systemsbiology.net/).^43^

### *pSBTetTA-YP_CFP* Transgene Construction

The plasmid *pSBtetTA-YP_CFP* was built from *pSBtet-GP* (Addgene #60495)^21^ as described in Supplemental Methods.

### Transgenic Cell Lines

DNA lipoplexes were formed with 1.3 μg *pSBtetTA-YP_CFP* plasmid, 0.1 μg helper SB100X plasmid^21^ (9:1 molar ratio of *pSBtetTA-YP_CFP* to SB100X), 5 μL Lipofectamine LTX, and 2.5 uL PLUS Reagent in Opti-MEM in a final volume of 500 μL. Lipoplexes for transient transfections did not include the helper SB100X plasmid. Lipoplexes were added dropwise to 2 × 10^5^ BT-549 or HEK293 cells in 2 mL growth medium without pen-strep in 6-well plates, and incubated for 2 days. Cells were harvested and expanded to 90% confluency in selection media (0.5–1 μg/mL puromycin) in 10 cm plates for about 6 days. Polyclonal transgenic cells were serial diluted 2-fold in a 6-well plate, and grown until single colonies were visible. Colonies were detached with 0.25% Trypsin buffer, transferred via micropipette into a 24-well plate with standard growth medium, and expanded to ∼90% confluency prior to outgrowth, LN2 storage, and downstream assays.

### Fluorescence Imaging with Microscopy

Live or fixed and stained cells were imaged in tissue culture plates on an EVOS M5000 inverted microscope (Thermo #AMF5000) at 40x, 100x or 200× magnification. Channel settings were as follows: RGB - no light cube, bright field; YFP - ex. 500/24, em. 542/27 (Thermo #AMEP4954); CFP - ex. 445/45, em. 510/42 (Thermo #AMEP4953). CFP turnover imaging is described in Supplemental Figure 4. Image processing was done with FIJI/ImageJ (version macOS x86_64).

### Flow Cytometry

At least 5 × 10^4^ cells were analyzed per run on a CytoFLEX Flow Cytometer with CytExpert software. Cells were harvested, washed with 1× DPBS, pelleted, and resuspended in FACS Buffer (10% FBS and 5 mM EDTA in 1× DPBS) on ice. Transgenic BT-549 cells treated with 0.5 μg/mL or no doxycycline were used as controls for CFP (K0525 V 525/40 Laser; ex. 468, em. 498) or YFP (FITC B 525/40 Laser ex. 498, em. 517), respectively. Parental cells were used as nonfluorescent controls for manually gating CFP or YFP-positive cells. Viability was verified with Zombie NIR (BioLegend #423105). Percent positive cells and mean fluorescence intensity (MFI) were determined using FlowJo software (version 10.1). Percent differences between UCM and ACM values for percent positive cells and for MFI were calculated as follows: (|average UCM values| – |average ACM values|)/|average UCM values| × 100.

### pSB Transgene Mapping

Cloning of genomic DNA fragments containing the junction between *pSBtetTA-YP_CFP* and adjacent genomic DNA into a customized vector (*pSBDest1GATC-Amp*) sequencing, and mapping of the transgene insertion site are described in Supplemental Figure 3.

### Real Time-Quantitative Polymerase Chain Reaction (RT-qPCR)

Total RNA was extracted using RNeasy Mini Kit (Qiagen #57801922) from pSBtetTA-YP_CFP BT-549 cells cultured in UCM or ACM + dox (0.5 μg/mL), or UCM without Dox (control) for 72 h. One μg RNA was extracted from two replicates (reps) per condition and used to synthesize cDNA (Invitrogen #18091050). qPCR was performed using PowerUp SYBR Green Master Mix (Applied Biosystems 100029284; Lot 01112585), 2 μL 1:1000 diluted cDNA, a 760 nM F/R primer mix, total volume 15 μL. Reactions were run on a QuantStudio 6 Flex Real-Time PCR System (Thermo #4484642) as follows: 1 cycle [50C, 2 min], 1 cycle [95C, 2 min] 40 cycles [95C, 15 s; 55–60C, 15 s; 72C, 1 min]. Gene targets and forward (fwd) and reverse (rev) primers were GAPDH (fwd 5′-TCT­CCT­CTG­ACT­TCA­ACA­GCG­AC; rev 5′-CCC­TGT­TGC­TGT­AGC­CAA­AT­TC), Tet1 (fwd 5′-GCA­AAG­TCA­TAA­ACG­GCG­CT, rev 5′-AGT­ACA­GGG­TAG­GCT­GCT­CA), Tet2 (fwd 5′-GAA­GGC­CTG­ACG­ACA­AGG­AA, rev 5′-ATC­TCG­ATT­GGC­AGG­GCA­TC), YFP1 (fwd 5′-CTA­CCC­CGA­CCA­CAT­GAA­GC; rev 5′-CTT­GTA­GTT­GCC­GTC­GTC­CT), YFP2 (fwd 5′-GCT­ACC­CCG­ACC­ACA­TGA­AG; rev 5′-TCT­TGT­AGT­TGC­CGT­CGT­CC), CFP1 (fwd 5′-ACA­TCC­TGT­CCA­CCG­TGT­TC; rev 5′-CTC­TCG­TAG­GAC­ATG­CCG­TC), CFP2 (fwd 5′-TCG­AGC­ACA­AGTC­CAC­CTTC; rev 5′-TCG­CAC­ACG­GTC­ATC­TTC­TC). GAPDH normalized values were calculated as dCt = average Ct_Target - average Ct_GAPDH (Target = Tet1, Tet2, YFP1, YFP2, CFP1, or CFP2). Control normalized values (ddCt) were calculated as 2^∧^-(dCt_Experimental replicate 1 - dCt_Control), where Control was UCM -dox rep. 1, and Experimental samples were UCM -dox rep. 1–3, UCM + dox rep. 1–3, or ACM + dox rep. 1–3.

## Supplementary Material



## Data Availability

The RNA-seq data is available as aligned paired-end reads (.fq.gz), counts in TPM and FPKM (.txt), and fold changes from ACM versus UCM (.txt) at NCBI GEO (accession GSE282461). Sequences for plasmids used in this study are available on Benchling: pSBtetTA-YP_CFP (https://benchling.com/s/seq-5G6pvKRBPlYj0l6zCuea?m=slm-yr0cAFTLoppZ139D8SK4), pSBDest1GATC-Amp (https://benchling.com/s/seq-GL2SdsnGw8rGrnBpabLl?m=slm-dL0Sug5Xn8NuG3DoHrl5).

## Notes

Supplemental figures showing promoter analysis of ACM-induced UpDEGs versus DownDEGs (Figure S1), additional BT-549 *pCMV-CFP* expression images (Figure S2, S7), the strategy for mapping the *pSBtetTA* insertion (Figure S3), episomal plasmid and lentiviral flow cytometry data (Figure S4), HEK293 flow cytometry data (Figure S5), additional RT-qPCR analysis (Figure S6), methods for culture media preparation, methods for transgene and vector construction, and supplemental references (PDF)
