# Supplementary material for: Tet Transgene Activation is Disrupted in Lipogenic Triple Negative Breast Cancer Cells
Source: ACS Synth Biol. 2025 Jul 8;14(7):2455–64. doi: 10.1021/acssynbio.4c00851 (PMC12281616; doi:10.1021/acssynbio.4c00851)
Supplement: Supplementary file 1 [file sb4c00851_si_001.pdf]

Supplemental information for

Tet Transgene Activation is Disrupted in Lipogenic Triple Negative Breast Cancer Cells

Ashley Townsel[1], Yifei Wu[2], Maya Jaffe[2], Cara Shields[1], Karmella A. Haynes[2]*

1. Department of Biology, Emory University, Atlanta, Georgia 30322, United States
2. Wallace H. Coulter Department of Biomedical Engineering, Emory University, Atlanta, Georgia 30322, United States

*Corresponding author: Karmella A. Haynes, kahayne@emory.edu

Supplemental Figures

- Figure S1. Comparison of the biological functions and promoters of UpDEGs versus DownDEGs.
- Figure S2. Comparison of the CFP-off state with CFP-on states in transgenic BT-549 cells.
- Figure S3. Isolation and sequencing of pSB-genomic DNA hybrid fragments from transgenic cells.
- Figure S4. Investigating effects of ACM treatment on different transgenic contexts: episomal plasmids and lentiviral inserts.
- Figure S5. Transgene expression in HEK293 cells treated with UCM or ACM.
- Figure S6. Additional RT-qPCR analysis of transgene mRNA levels in uninduced (UCM), and doxycycline-induced cells treated with UCM or ACM.
- Figure S7. CFP signal turnover after *pCMV-CFP* transcription is stopped by inducer washout.

Supplemental Methods

- Preparation of Culture Media
- *pSBTetTA-YP_CFP* Transgene Construction.
- *pSBDest1GATC-Amp* Vector Construction

SUPPLEMENTAL FIGURES

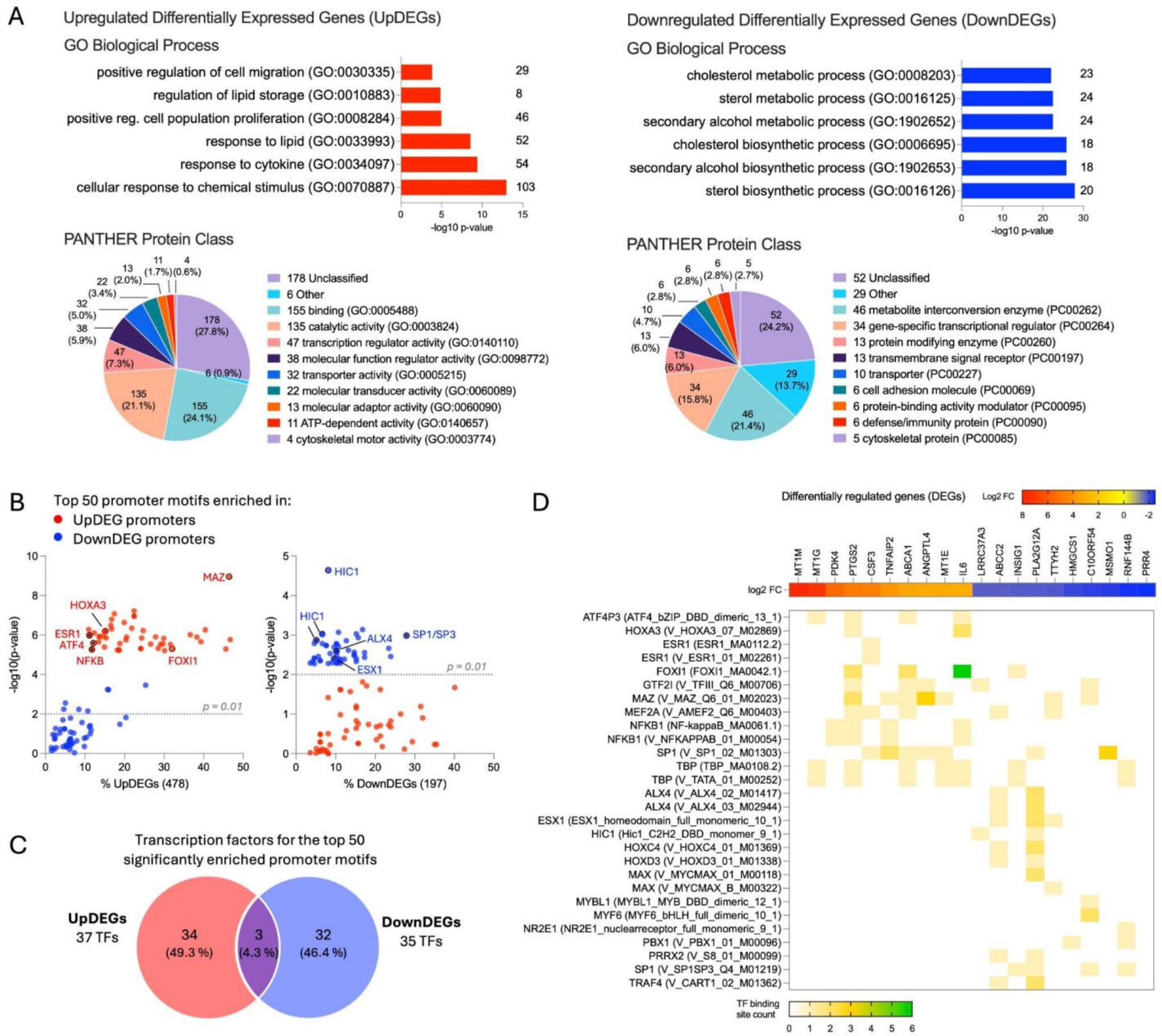

Figure S1. Comparison of the biological functions and promoters of UpDEGs versus DownDEGs. (A) Panther (Release 19.0 [1]) was used to determine GO Biological Process enrichments (overrepresentation test, Fisher's Exact) and PANTHER protein class for 495 UpDEGs and 210 DownDEGs. (B) The script TF_targets was downloaded from https://github.com/cplaisier/TF_targets and used to find enriched transcription factor binding motifs in DEGs represented in the TFBS database (478 UpDEGs, 197 DownDEGs) [2]. (C) The Venn diagram compares transcription factors that bind UpDEG or DownDEG enriched promoter motifs from B. (D) Occurrences of promoter motifs from B that appeared almost exclusively in the promoters of the top 10 UpDEGs or DownDEGs. Transcription factors that recognize select motifs from this chart are labeled in the dotplots in B.

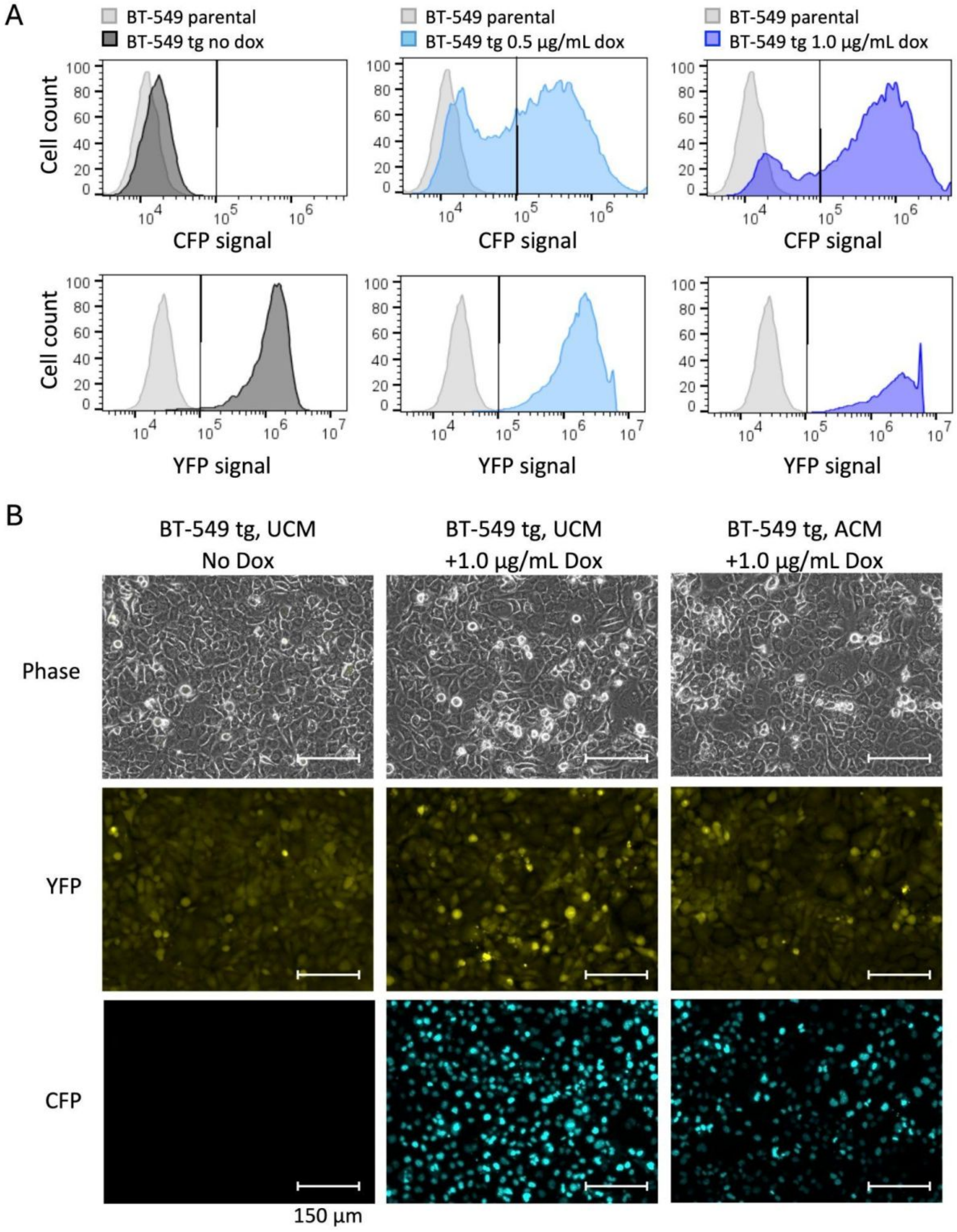

Figure S2. Comparison of the CFP-off state with CFP-on states in transgenic BT-549 cells. (A) To determine the dynamic range of doxycycline (dox) mediated regulation of CFP-NLS, transgenic (tg) cells were seeded in 6-well plates, grown in unconditioned medium (UCM) without dox, with 0.5 µg/mL Dox, or 1.0 µg/mL Dox for two days and analyzed by flow cytometry. (B) Cells were grown in unconditioned medium (UCM), UCM plus 1.0 µg/mL doxycycline (Dox), or adipocyte conditioned medium (ACM) plus 1.0 µg/mL Dox and imaged after two days.

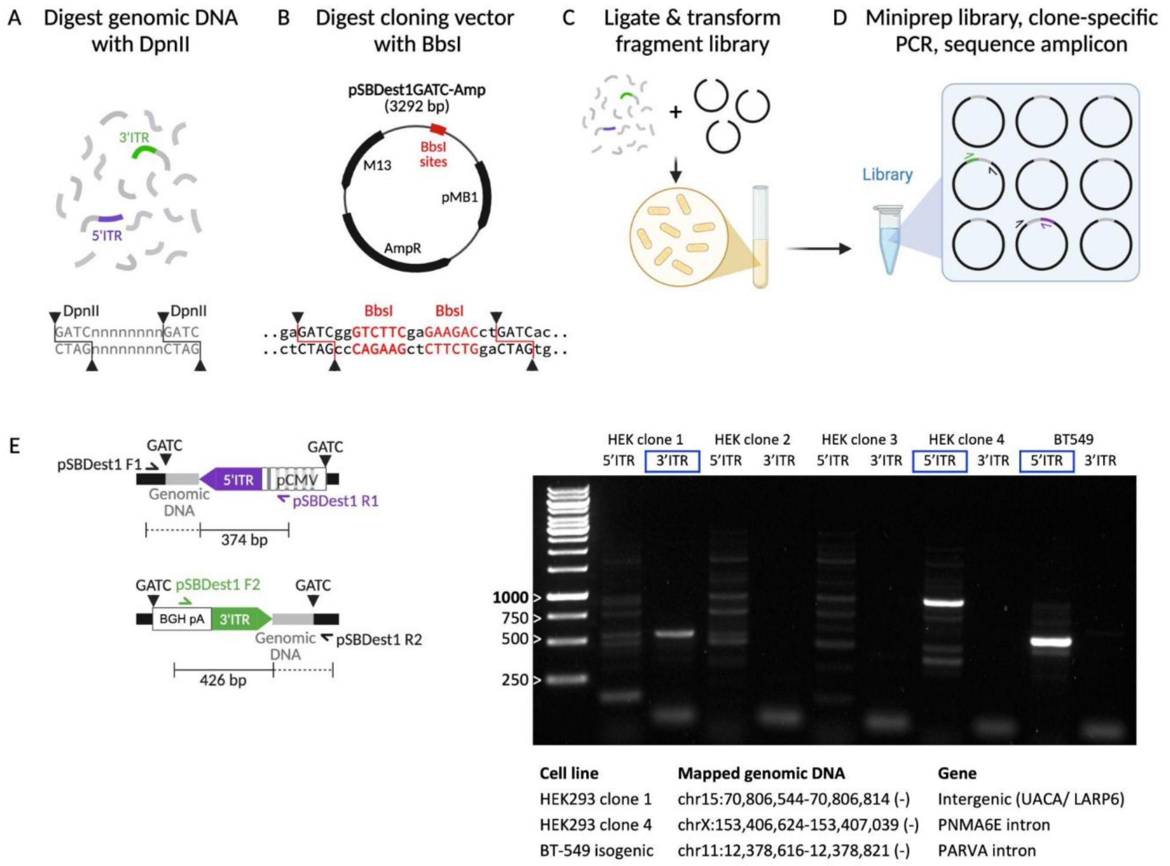

Figure S3. Isolation and sequencing of pSB-genomic DNA hybrid fragments from transgenic cells. (A) 600 ng of genomic DNA from transgenic cells (Genelute kit, Sigma #G1N70) was digested and dephosphorylated with FD DpnII (MboI) (Thermo #FD0814), Quick CIP (NEB #M0525S), and 10X buffer (Thermo #FERB64) in a final volume of 30 µL. (B) Plasmid pSBDest1GATC-Amp was built as described in Supplemental Methods. (C) DNA digests (3 µL, 60 ng DNA) were heat-deactivated and ligated with 50 ng BbsI-digested, column-purified pSBDest1GATC-Amp in 1X T4 ligase buffer (NEB #B0202S) with 1 µL T4 Quick Ligase (NEB #M2200S), in a final volume of 10 µL. 50 µL DH5-α Turbo (NEB #C2984) cells were transformed with 10 µL ligation reaction, incubated on ice for 5 min, added to 3 mL LB broth (100 mg/mL ampicillin), and grown with shaking overnight at 37°C. (D, E) Libraries were purified using a miniprep kit (Zymo D4016) and used for PCR: pSBDest1 F1 (5'-gacgttgtaaaacgacggccag) and pSBDest1 R1 (5'-cactgatagggagtaaactcgacatcg); or pSBDest1 F2 (5'-catcgcattgtctgagtaggtgtc) and pSBDest1 R2 (5'-acaatttcacacaggaaacagctatgac); 10 ng library miniprep, 1 µL each 10 µM primer, 1X DreamTaq PCR master mix (Thermo #K1081), final volume 50 µL; 95°C, 1 min; 35x [95°C, 15 sec; 57°C, 15 sec; 72°C, 40 sec]; 72°C, 3 min; 4°C hold. 3 µL of each product resolved on a 1% agarose/ 1X TAE/ 1X SYBR Safe gel with 3 µL GeneRuler 1 Kb (Thermo #SMO314, lane 1). PCR products were gel purified (NEB #T1020S), and analyzed by Sanger sequencing (Azenta Genewiz). Non-vector sequence adjacent to the GATC ligation site was mapped using BLAT in the Integrative Genomics Viewer (IGV) [3].

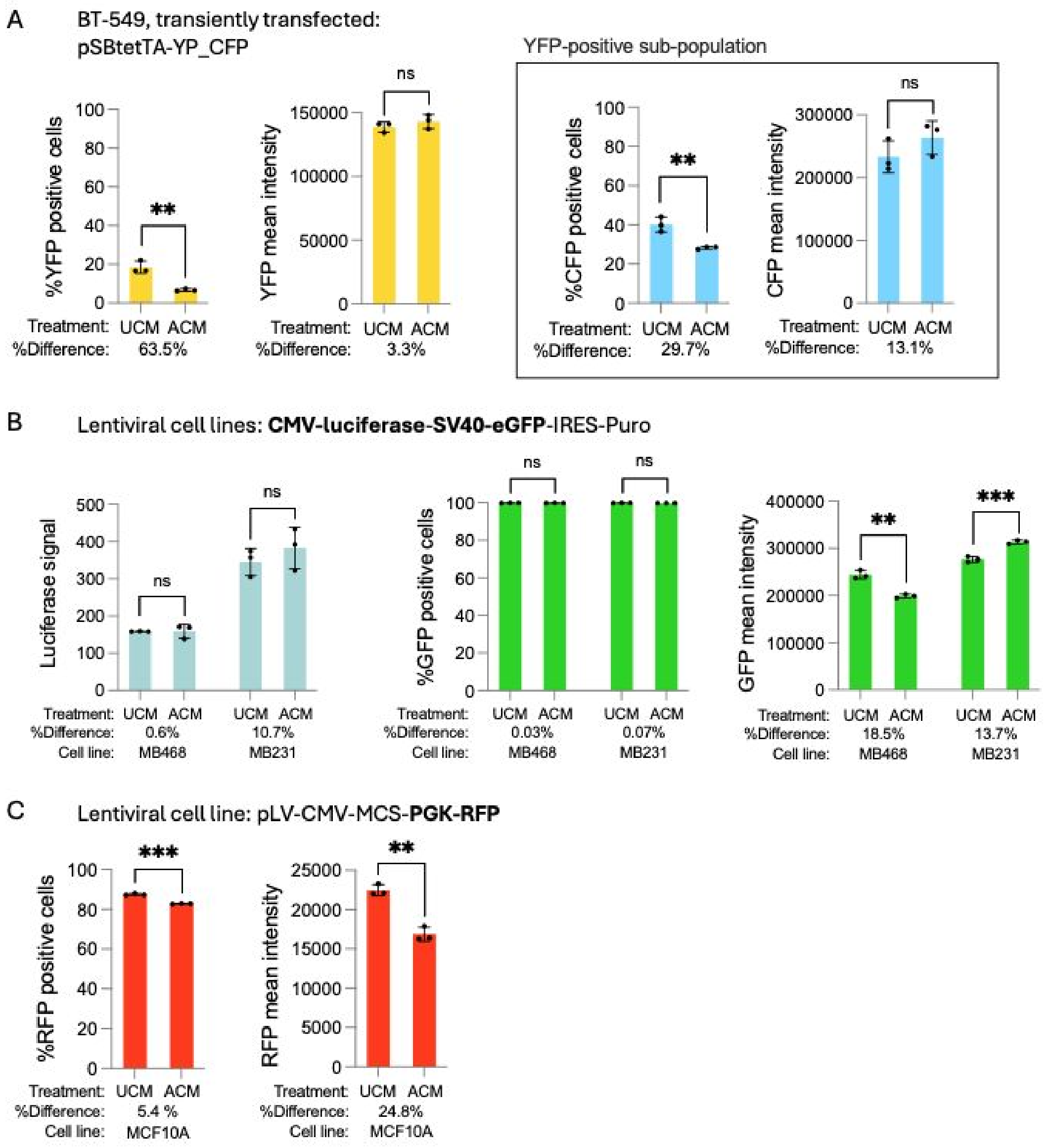

Figure S4. Investigating effects of ACM treatment on different transgenic contexts: episomal plasmids and lentiviral inserts. (A) Cells were transfected with 1 µg pSBtetTA-YP_CFP, without SB100X, and cultured for 2 days. Cells were treated with UCM or 75% ACM for 3 days and harvested for flow cytometry (B) Lentiviral breast cancer cells carrying CMV-luciferase-SV40-eGFP-IRES-Puro (MDA-MB-468 and MDA-MB-231) or pLV-CMV-MCS-PGK-RFP (MCF-10A) were treated with 1ug/mL Puromycin and UCM or 75% ACM for 3 days. For flow cytometry, cells were harvested and fixed with 4% paraformaldehyde. For luciferase assays, approximately 2x10^4 cells were lysed using the Pierce Firefly Luciferase Glow Assay Kit (Thermo Scientific #16176) and luminescence was measured with a BioTek Synergy LX plate reader. Bar charts show means of three replicate wells per condition, percent differences for mean UCM versus mean ACM, standard deviation (black bars), and unpaired t-test values: $p \leq 0.05$*, 0.01**, 0.001***, or not significant (ns).

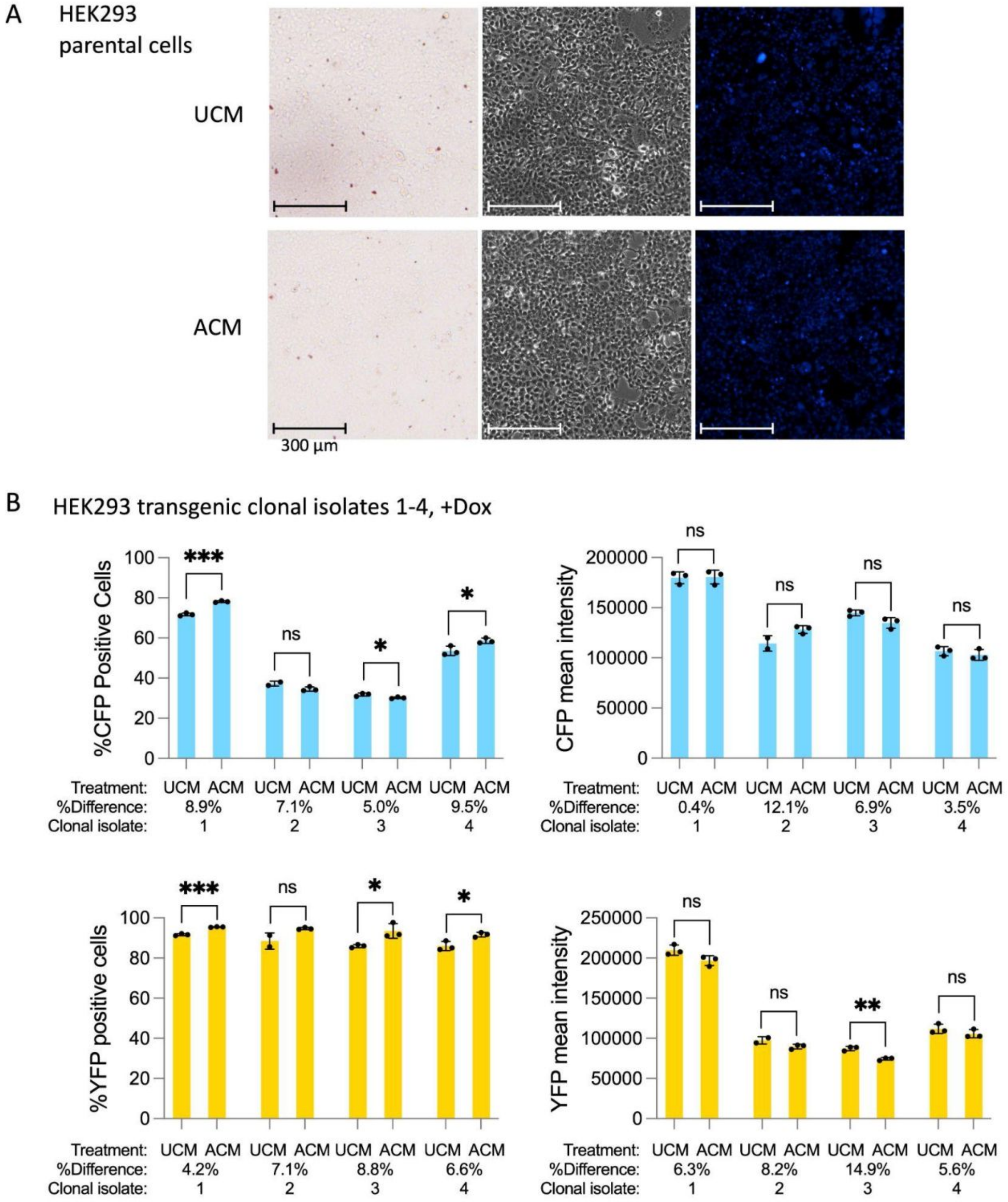

Figure S5. Transgene expression in HEK293 cells treated with UCM or ACM. (A) Oil Red O staining of HEK293 parental cells grown in UCM or ACM medium showed very little visible lipid droplet formation and no increase in staining in ACM-treated cells. Nuclei in transgenic HEK cells were visualized by counterstaining with 300 µM DAPI (Bioquest #17507). Images were taken with a BioTek Lionheart FX microscope. (B) Bar charts show means of three replicate wells per condition, percent differences for

mean UCM versus mean ACM, standard deviation (black bars), and unpaired t-test values: $p \leq 0.05$*, 0.01**, 0.001***, or not significant (ns).

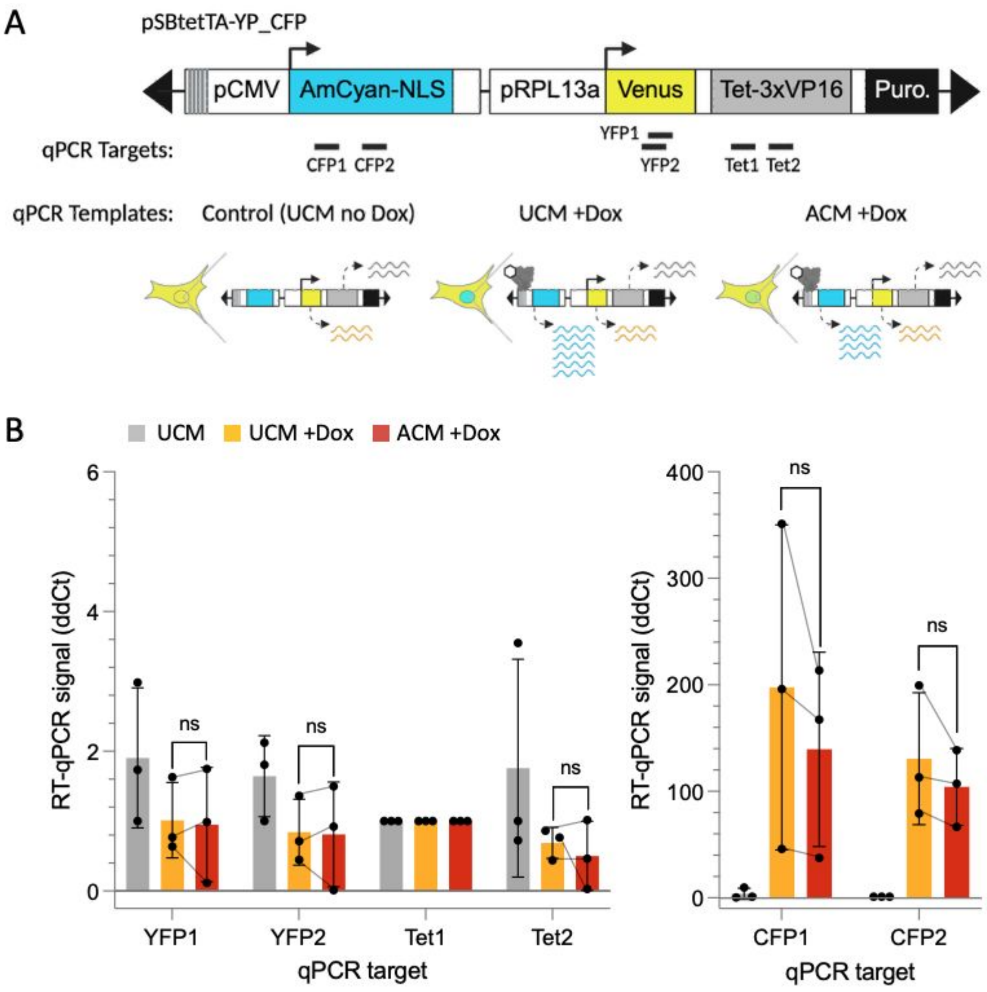

Figure S6. Additional RT-qPCR analysis of transgene mRNA levels in uninduced (UCM), and doxycycline-induced cells treated with UCM or ACM. (A) Two different sites were targeted in each open reading frame: the CFP region of CFP-NLS (CFP1, CFP2), YFP (YFP1, YFP2), and the Tet region of Tet-TA (Tet1, Tet2). (B) Values were normalized with Tet1 as an internal reference. Tet1 normalized values were calculated as dCt = average Ct_Tet1 - average Ct_Target (Tet2, YFP1, YFP2, CFP1, or CFP2). Control normalized values (ddCt) were calculated as 2^-(dCt_Control replicate 1 - dCt_Experimental), where Experimental samples were UCM -dox rep. 1-3, UCM +dox rep. 1-3, or ACM +dox rep 1-3. P-values were calculated with a paired two-tailed t-test: $p \leq 0.05$*, 0.01**, 0.001***, or not significant (ns).

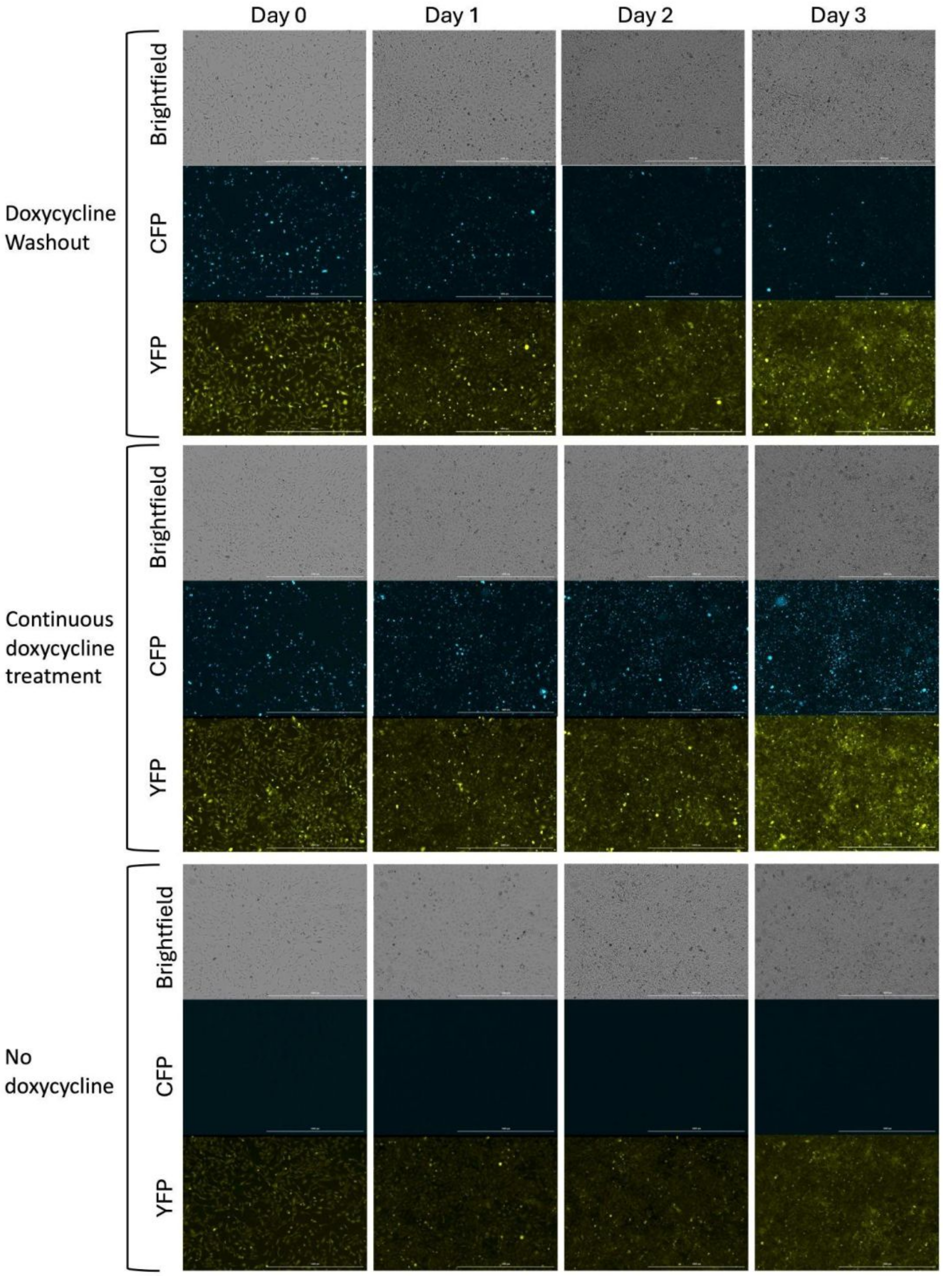

Figure S7. CFP signal turnover after *pCMV-CFP* transcription is stopped by inducer washout. Previously reported half-lives of fluorescent proteins in human cells range from 4 - 14 hours [4]. Clonal BT-549 pSBtetTA-YP_CFP cells were seeded at 100,000 cells per well in a 6-well plate and grown for 5 days. "Doxycycline washout" and "continuous doxycycline treatment" groups were treated with 0.5 µg/mL doxycycline in complete BT-549 medium for 2 days, then imaged (Day 0). The media was replaced and the "continuous doxycycline treatment" group was given 0.5 µg/mL doxycycline. The cells were imaged on Day 1, 2, and 3 using the Brightfield, CFP (ex. 445/45; em. 510/42), and YFP (ex. 500/24; em. 542/27) channels in a BioTek Cytation 1 Image Reader (Fisher Scientific #CYT1F4AV). Scale bars = 1000 µm.

SUPPLEMENTAL METHODS

Preparation of Cell Culture Media

Formulas for the cell lines used in this study are described in the table below. Catalog numbers for media reagents are as follows: RPMI-1640 (ATCC #30-2001), DMEM High Glucose (Thermo #11965092), alpha-MEM (Gibco #12561056), DMEM (Hyclone #SH30243.01), standard fetal bovine serum (FBS, Bio-Techne #S11150), tetracycline system approved FBS (tsFBS, Thermo #A4736401), insulin (Thermo #12585014), penicillin-streptomycin (Thermo Fisher #15140122), sodium oleate (Sigma #O7501), fatty acid free bovine serum albumin (BSA, Sigma #A6003).

Cell line (catalog no.)	Base medium	Formula
BT-549 (ATCC #HTB-122)	RPMI-1640	10% tsFBS, 0.8 ug/mL insulin
HCC1806 (ATCC #CRL-2335)	RPMI-1640	10% tsFBS, 1% penicillin-streptomycin, 1% L-glutamine, 1% Sodium Pyruvate
MDA-MB-453 (ATCC #HTB-131)	RPMI-1640	10% tsFBS
HEK293 (ATCC #CRL-1573)	DMEM high glucose	10% tsFBS
OP9 (ATCC #CRL-2749)	alpha-MEM	20% FBS, 1% penicillin-streptomycin
OP9: stromal cell propagation	DMEM	10% FBS, 1% penicillin-streptomycin
OP9: differentiation IOM	alpha-MEM	0.2% FBS, 1.8% BSA (in 1X PBS), 1.52 mM sodium oleate (in methanol), 175 nM insulin, 1% penicillin-streptomycin

Preparation of insulin oleate medium (IOM) for OP9 differentiation. A 100 mM stock solution of Sodium Oleate was prepared with 100% methanol, and a stock solution of 30% fatty acid free BSA was prepared with 1X PBS. Each solution was passed through a 0.22 µm filter before use. The Insulin Oleate Media (IOM) culture media was prepared with 1.52 mM sodium oleate (Sigma #O7501) and 1.8% fatty acid free BSA (Sigma #A6003) and incubated at 37°C for 2 hours. After incubation, the IOM media was supplemented with 0.2% FBS, 175 nM insulin, and 1% penicillin-streptomycin (Thermo Fisher Scientific #15140122). The final volume of IOM was made up of alpha-MEM media.

Preparation of adipocyte-conditioned media (ACM). OP9 cells were seeded at a density of $5x10^5$ and cultured in OP9 stromal propagation medium for 24 hours. The medium was replaced with 10 mL IOM, cells were cultured for 72 hours, and the supernatant adipocyte-

conditioned media (ACM) was collected and centrifuged at room temperature in a 50 mL conical tube at 1200 rpm (270 x g) for 5 minutes before storage as 6 mL aliquots in a -20°C freezer. ACM was diluted to 50% with unconditioned medium (UCM) and used for the Oil Red O staining and the cluster analysis of polygenic BT-549 cells, while 100% ACM was used for the RNA-seq experiments. ACM was diluted to 75% ACM and used for all other experiments with transgenic cells.

pSBTetTA-YP_CFP **Transgene Construction**

The plasmid *pSBtetTA-YP_CFP* was built from *pSBtet-GP* (Addgene #60495) [5]. An EcoRI site was generated (5'-gag to gaa) at eGFP E223 via site directed mutagenesis (NEB #E0554) with primers 5'-tcctgctggaAttcgtgaccg and 5'-ccatgtgatcgcgcttctcg. A Eco81I/ EcoRI-flanked Yellow Fluorescent Protein (YFP) fragment (M plus V1..L221) was generated from Venus BBa_J176006 with primers 5'-tctgcacctgaggccaccatggtgagcaagggcgagg and 5'-ggtcacgaattccagcaggaccatgtgatcg via high fidelity PCR (NEB #E0555) followed by spin-column purification (Sigma #NA1020). The YFP fragment and mutated pSBtet-GP plasmid were double-digested with Eco81I/ EcoRI (Thermo #FD0374, #FD0274), gel-purified (NEB #T1020), and ligated (NEB #M2200 with 10x ligase buffer NEB #B0202S) to build *pSBtetTA-YP_luc*. An SfiI-flanked CFP+NLS fragment (M1..R231 plus nuclear localization signal PKKKRKV) was generated from AmCyan-NLS in BBa_S04698 with primers 5'-tgaaggcctctgaggccaccatggcgctgtccaacaag and 5'-gcttggcctgacaggccttataccttgcgctttttcttggg via high fidelity PCR followed by spin-column purification. SfiI-digested CFP+NLS was ligated into SfiI-linearized *pSBtetTA-YP* to replace luciferase. Transformations were done with NEB Turbo DH5-α cells (NEB #C2984) in 100 µg/mL ampicillin selection media without heat shock or recovery.

pSBDest1GATC-Amp **Vector Construction**

To build vector pSBDest1GATC-Amp, a “BbsI sites” DNA fragment with XbaI and PstI overhangs was built from annealed oligos: 10 µM 5'-CTAGaGATCggGTCTTCgaGAAGACctGATCactagtagcggccgcTGCA, 10 µM 5'-gcggccgctactagtGATCagGTCTTCtcGAAGACccGATCt, 1X T4 ligation buffer (NEB #B0202S), and T4 PNK (NEB #M0201) in a final volume of 10 µL; thermal cycler program: 37°C (30 min), 95°C (5 min), ramp-down at 5°C/ min (0.1°C/ sec) to 25°C. The dsOligo product was ligated with XbaI/ PstI-linearized, column-purified GGDest1-Amp (Addgene #157649): 1 µL 1:250 diluted

dsOligo, 25 ng linearized GGDest1-Amp, 1X T4 ligase buffer (NEB #B0202S), µL T4 Quick Ligase (NEB #M2200S), final volume 10 µL. Transformations were done with NEB Turbo DH5-α cells (NEB #C2984) in 100 µg/mL ampicillin selection media without heat shock or recovery.

SUPPLEMENTAL REFERENCES

(1) Thomas, P. D.; Ebert, D.; Muruganujan, A.; Mushayahama, T.; Albou, L.-P.; Mi, H. PANTHER: Making Genome-Scale Phylogenetics Accessible to All. *Protein Sci.* **2022**, *31* (1), 8–22.

(2) Plaisier, C. L.; O'Brien, S.; Bernard, B.; Reynolds, S.; Simon, Z.; Toledo, C. M.; Ding, Y.; Reiss, D. J.; Paddison, P. J.; Baliga, N. S. Causal Mechanistic Regulatory Network for Glioblastoma Deciphered Using Systems Genetics Network Analysis. *Cell Syst* **2016**, *3* (2), 172–186.

(3) Robinson, J. T.; Thorvaldsdóttir, H.; Winckler, W.; Guttman, M.; Lander, E. S.; Getz, G.; Mesirov, J. P. Integrative Genomics Viewer. *Nat. Biotechnol.* **2011**, *29* (1), 24–26.

(4) Chen, W.; Smeekens, J. M.; Wu, R. Systematic Study of the Dynamics and Half-Lives of Newly Synthesized Proteins in Human Cells. *Chem Sci* **2016**, *7* (2), 1393–1400.

(5) Kowarz, E.; Löscher, D.; Marschalek, R. Optimized Sleeping Beauty Transposons Rapidly Generate Stable Transgenic Cell Lines. *Biotechnol. J.* **2015**, *10* (4), 647–653.
